# Induction of Cell Cycle Arrest, Apoptosis, and Reducing the Expression of MCM Proteins in Human Lung Carcinoma A549 Cells by Cedrol, Isolated from *Juniperus chinensis*

**DOI:** 10.4014/jmb.2205.05012

**Published:** 2022-07-01

**Authors:** Hee Jung Yun, Da Jeoung Jeoung, Soojung Jin, Jung-ha Park, Eun-Woo Lee, Hyun-Tai Lee, Yung Hyun Choi, Byung Woo Kim, Hyun Ju Kwon

**Affiliations:** 1 Biopharmaceutical Engineering Major, Division of Applied Bioengineering, College of Engineering, Dong-eui University, Busan 47340, Republic of Korea; 2 Department of Biopharmaceutics, Dong-eui University Graduate School, Busan 47340, Republic of Korea; 3 Core-Facility Center for Tissue Regeneration, Dong-eui University, Busan 47340, Republic of Korea; 4 Department of Biochemistry, College of Korean Medicine, Dong-eui University, Busan 47227, Republic of Korea; 5 Blue-Bio Industry Regional Innovation Center, Dong-eui University, Busan 47340, Republic of Korea

**Keywords:** Apoptosis, cedrol, cell cycle arrest, *Juniperus chinensis*, lung carcinoma A549, minichromosome maintenance proteins

## Abstract

Proteins related to DNA replication have been proposed as cancer biomarkers and targets for anticancer agents. Among them, minichromosome maintenance (MCM) proteins, often overexpressed in various cancer cells, are recognized both as notable biomarkers for cancer diagnosis and as targets for cancer treatment. Here, we investigated the activity of cedrol, a single compound isolated from *Juniperus chinensis*, in reducing the expression of MCM proteins in human lung carcinoma A549 cells. Remarkably, cedrol also strongly inhibited the expression of all other MCM protein family members in A549 cells. Moreover, cedrol treatment reduced cell viability in A549 cells, accompanied by cell cycle arrest at the G1 phase, and enhanced apoptosis. Taken together, this study broadens our understanding of how cedrol executes its anticancer activity while demonstrating that cedrol has potential application in the treatment of human lung cancer as an inhibitor of MCM proteins.

## Introduction

When DNA mutations accumulate or damaged DNA is not repaired, the integrity of the genome is disrupted, leading to cancer or cancer-causing diseases. Therefore, replication-related proteins or factors involved in DNA repair can be targets for anticancer drugs. Minichromosome maintenance (MCM) proteins, a group of regulatory proteins essential for the initiation and elongation of eukaryotic DNA replication, are closely related to cancer formation and are recognized as notable biomarkers for diagnosing cancer and as targets for cancer treatment [1]. The MCM proteins have six subunits (*i.e.*, MCM2 to MCM7) forming a heterohexameric complex, which acts as a component of the pre-replicative complex at the replication origin in the early G1 phase and as a helicase at the replication fork between the late G1 and S phases [2, 3]. The MCM complex is also a replication licensing factor that allows only one DNA replication to occur per cell cycle. It has been reported that the decrease in the expression of MCM proteins is associated with the activation of checkpoints, which is an important regulatory factor for cell cycle progression: knockdowns of MCM2, MCM3, MCM6, and MCM7 induced cell cycle arrest at the G1/S or G2/M, G1, S/G2, and G1/S phases, respectively [4-10]. In addition, the MCM genes are overexpressed in both cancer tissues in vivo and cancer cell lines in vitro, and the inhibition of cell proliferation has been reported in many MCM knockdown cancer cells [6, 11, 12]. Thus, the MCM proteins can be a potential diagnostic marker for cancer cells (or cells that are likely to become cancer cells) and a target for cancer treatment.

Molecules that reduce the expression of proteins constituting the MCM complex or inhibit the helicase enzyme activity of the MCM complex were reported in a previous study aimed at developing new anticancer agents [13]. Trichostatin A (TSA), a classical histone deacetylase inhibitor, and genistein, a nontoxic dietary isoflavone isolated from *Genista tinctoria*, inhibited the expression of MCM2 and MCM2-7 loading factors, such as cyclin-dependent kinase 2 (CDK2), in prostate cancer [14]. TSA induced apoptosis in HCT116 cells through the activation of the JNK pathway, which appeared to be involved in the decrease in the expression of MCM2. Lovastatin, an inhibitor of 3-hydroxy-3-methylglutaryl coenzyme A reductase, also inhibited the MCM2 expression through the JNK pathway and induced G1/S arrest and apoptosis [8]. Atorvastatin, another statin drug, decreased the expression of MCM6 and MCM7 [15]. Metformin, which inactivates mTOR and induces cell cycle arrest in colorectal cancer cells, inhibited the expression of cell cycle regulatory proteins including MCM2 [16]. Breviscapine, a natural flavonoid, inhibited MCM7 and induced apoptosis in human prostate cancer cells [17]. Some antibiotics, including heliquinomycin and ciprofloxacin, have been reported to inhibit the helicase enzyme activity of the MCM complex [18-21].

Numerous studies on crude extracts and/or bioactive compounds isolated from natural products used in traditional medicine have shown these materials to have great promise in the treatment of cancer. Obviously, many natural plant-derived drugs such as paclitaxel, vinblastine, vincristine, and camptothecin have been applied as highly effective anticancer agents [22, 23]. Our research team has been searching for substances that regulate the expression of MCM proteins in natural products. As a result, it was reported that widdrol, a fragrance component isolated from *Juniperus chinensis*, inhibited the expression of MCM proteins. Widdrol induced cell cycle arrest, apoptosis [24, 25], and activation of DNA damage checkpoint [26] in colon cancer HT29 cells. In addition to widdrol, cedrol, another fragrance component isolated from *J. chinensis*, also showed inhibitory activity on MCM protein expression. In this study, therefore, we report that cedrol isolated from *Juniperus chinensis* inhibited the expression of MCM proteins and cell viability, induced cell cycle arrest and apoptosis, and altered the expression of cell cycle-related proteins and apoptosis-related proteins in human lung carcinoma A549 cells.

## Materials and Methods

### Cell lines and Culture

Cell lines of human lung carcinoma A549 were purchased from American Type Culture Collection (USA) and grown in RPMI 1640 medium supplemented with 10% (v/v) fetal bovine serum (heat inactivated) and 1%penicillin/streptomycin. Cells were incubated at 37°C in humidified conditions of 5% CO_2_ to continue growth and proliferation.

### Bioassay-Guided Fractionation for *Juniperus chinensis*

Dried and ground *J. chinensis* (10 kg) was refluxed with methanol (repeated 3 times) at 75°C for 4 h. The whole filtrate was concentrated to dryness in vacuo at 40°C to obtain a viscous syrup, which was then lyophilized to obtain a powdered methanol extract (635.5 g). Subsequent bioassay-guided fractionations of the methanol extract were performed to obtain a single active compound. The powdered methanol extract was suspended in distilled water (DW) containing 10% methanol, and sequentially partitioned with dichloromethane (CH_2_Cl_2_, 301.8 g), ethyl acetate (EtoAc, 156.0 g), n-butanol (n-BuOH, 133.5 g) and aqueous (H_2_O, 44.2 g) in sequence. The active CH_2_Cl_2_ fraction was chromatographed over a silica gel column, thereby yielding 32 fractions (*i.e.*, F1-F32). The most active fraction 9 (F9) was further purified by recrystallization to obtain a pure single compound (123.8 g).

### Western Blot Analysis

Cells were lysed in cytoskeletal (CSK) buffer {10 mM piperazine-*N,N*’-bis(2-ethanesulfonic acid, pH 6.8, 100 mM NaCl, 1 mM MgCl_2_, 1 mM EGTA, 1 mM dithiothreitol, 1 mM phenylmethanesulfonyl fluoride, 0.1%Triton X-100, 1 mM ATP, and complete protease inhibitor cocktail (BD Pharmingen, USA)} at 4°C for 15 min. Cell lysates were disrupted by sonication and centrifuged at 20,000 ×*g* for 30 min, and the protein concentration of the supernatant was determined using a BCA Protein Assay Kit (Bio-Rad, USA). Equal amounts of proteins were separated on SDS-polyacrylamide gels and transferred onto polyvinylidene fluoride membranes (Pall Corporation, USA). The membranes were incubated with primary antibodies at 37°C for 1 h in blocking solution (Blokace, Dai-Nippon, Japan), washed with Tris-buffered saline (50 mM Tris/HCl, pH 7.5, 0.15 M NaCl, and 0.1% Triton X-100), and incubated with peroxidase-conjugated secondary antibodies (Pierce, USA). All antibodies used in this study were purchased from Santa Cruz Biotechnology (USA), with the exception of antibodies to cleaved caspase-3, Bax, and Bcl-2, which were purchased from Cell Signaling Technology (USA). The immunoreacted proteins were detected using a chemiluminescence system (SuperSignal West Femto Maximum Sensitivity Substrate, Thermo Fisher Scientific, USA), and their reactivity was quantified using a Fluorchem™ 5500 (Alpha-InnoTech, USA).

### Cell Viability Assay

Cell viability was measured using a water-soluble, tetrazolium salt-based EZ-Cytox Cell Viability Assay Kit (DoGenBio, Korea) and the experimental procedure followed the manufacturer’s manual. Cells were seeded in 96-well plates at a density of 1 × 10^4^ cells/well, incubated at 37°C for 24 h, and then treated with cedrol at various concentrations. After an additional 48 h incubation at 37°C, 10 μl of EZ-Cytox assay reagent was added to each well and the plates were incubated at 37°C for an additional 10 min. Absorbance values at 450 nm were measured using a microplate reader for the calculation of cell viability. The percentage of cell viability was calculated by the following formula: % cell viability = (mean absorbance of test wells)/(mean absorbance of control wells) × 100.

### Cell Cycle Analysis

Cell cycle profiles were evaluated using a Muse Cell Cycle Kit with a Muse Cell Analyzer (Merck Millipore, USA) according to the manufacturer’s protocol at the Core-Facility Center for Tissue Regeneration, Dong-eui University (Korea). Cells were seeded at a density of 1 × 10^5^ cells/well in 6-well plates, incubated at 37°C for 24 h, and then treated with various concentrations of cedrol. After an additional 48 h incubation at 37°C, around 1×10^6^ cells were centrifuged at 300 ×*g* for 5min. Cells in the pellet were washed twice with phosphate-buffered saline and fixed with 70% ethanol at -20°C for 3h. Following that, 200 μl of fixed cells and an equal volume of Muse cell cycle reagent were mixed and incubated for 30min at room temperature in the dark. The cell cycle was then analyzed using the above-mentioned Muse Cell Cycle Kit and Muse Cell Analyzer.

### Cell Apoptosis Assay

Cell apoptosis was measured using a Muse Annexin V and Dead Cell Assay Kit (Merck Millipore) according to the user’s guide. A549 cells were seeded in 6-well plates at an initial density of 1 × 10^5^ cells/well. After 24 h of culture, cells were treated with various concentrations of cedrol for 48 h. A total of 1 × 10^5^ cells were washed with phosphate-buffered saline, resuspended in medium with 1% bovine serum albumin and 10% fetal bovine serum, mixed with Muse Annexin V and Dead Cell reagent, and then incubated for 20 min at room temperature in the dark. Results were analyzed using a Muse Cell Analyzer at the Core-Facility Center for Tissue Regeneration, Dong-eui University (Korea).

### Statistical Analysis

Data were expressed as the mean ± SD and evaluated by one-way analysis of variance followed by the unpaired Student’s *t*-test as a post hoc analysis. Differences were regarded as significant when the *p*-values were less than 0.05.

## Results

### Cedrol Was Isolated from *J. chinensis*

**Fig. 1:**
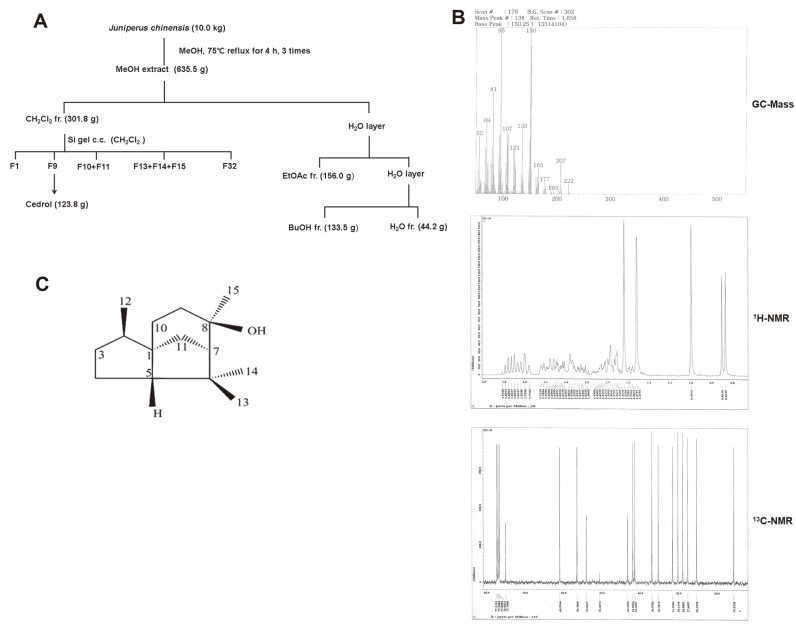
Isolation and identification of cedrol from *J. chinensis*. (**A**) Extraction and fractionation procedure of *J. chinensis* to obtain cedrol. (**B**) GC-Mass, ^1^H-NMR, and ^13^C-NMR spectra of cedrol. (**C**) Molecular structure of cedrol.

Bioassay-guided fractionation and isolation on *J. chinensis* was performed to obtain a single active compound identified as cedrol (Fig. 1). The overall extraction and fractionation procedure of *J. chinensis* is shown in Fig. 1A. Compound 1 (cedrol) from *J. chinensis* was obtained as a white powder and produced a molecular ion peak at *m/z* 222 in the EI-MS spectrum (Fig. 1B). The molecular formula was assigned as C_15_H_2_6O based on ^13^C-NMR data (Fig. 1B) and the molecular ion peak in EI-MS. The ^1^H-NMR spectrum (Fig. 1B) showed 4 methyl singlets at δ 0.84, 0.99, 1.26, and 1.32. Fifteen carbon signals appeared in the ^13^C-NMR spectrum, including the hydroxyl group at δ 75.1 and a tetrasubstituted methyl group (δc 15.5, 27.6, 28.9 and 30.1). The above data suggested that the structure of compound 1 was cedrol (Fig. 1C).

Cedrol: white needless, EIMS *m/z*: 222 [M]^+^, ^1^H-NMR (400 MHz, CD3OD): δ 0.84 (3H, d, *J* = 6.9 Hz, CH_3_-12), 0.99 (3H, s, CH_3_-13), 1.26 (3H, s, CH_3_-14), 1.32 (3H, s, CH_3_-15), ^13^C-NMR (100 MHz, CD3OD): δ54.1 (C-1), 41.4 (C-2), 37.0 (C-3), 25.3 (C-4), 61.0 (C-5), 43.4 (C-6), 56.5 (C-7), 75.1 (C-8), 35.3 (C-9), 31.6 (C-10), 42.0 (C-11), 15.6 (C-12), 27.6 (C-13), 28.9 (C-14), 30.1 (C-15).

### Cedrol Inhibited Cell Proliferation and Expression of MCM Proteins

**Fig. 2:**
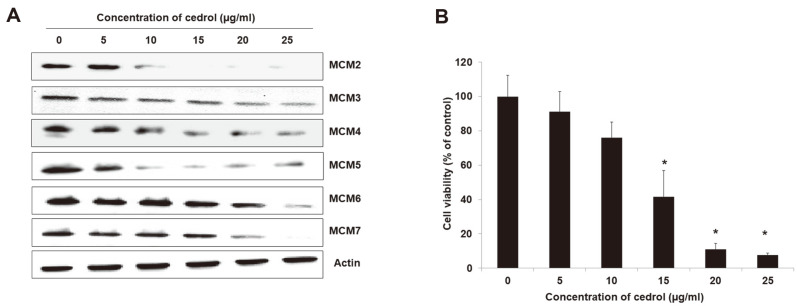
Cedrol inhibits the expression of MCM proteins and cell proliferation in human lung carcinoma A549 cells. (**A**) Western blot analysis was performed after treatment with cedrol at the indicated doses (*i.e.*, 0, 5, 10, 15, 20, and 25 μg/ ml) for 48 h. Actin was used as an internal control. (**B**) After treatment with indicated doses of cedrol for 48 h, cell viability was measured using an EZ-Cytox Cell Viability Assay Kit and expressed as a percentage of control. Data are presented as mean ± SD of three independent experiments. *p < 0.05 vs. each control.

To confirm that cedrol isolated from *J. chinensis* inhibits the expression of MCM proteins in A549 cells, western blot analysis was performed after treatment with various concentrations of cedrol (0, 5, 10, 15, 20, and 25 μg/ml) on A549 cells for 48 h (Fig. 2A). As a result, the expression of all MCM proteins was reduced in A549 cells treated with cedrol in a dose-dependent manner. As an essential replication factor, MCM proteins are closely related to cell proliferation and can be used as a diagnostic marker for cell proliferation [5, 6]. Thus, we examined the effect of cedrol on the viability of A549 cells (Fig. 2B). A549 cells were treated with cedrol for 48 h and viable cells were measured using an EZ-Cytox Cell Viability Assay Kit. Compared to control cells, cell viability was decreased upon treatment with cedrol in A549 cells in a dose-dependent manner. Especially, the cell viability was significantly (*p* < 0.05) decreased at the concentration of 15~25 μg/ml cedrol, where all MCM expression was significantly reduced.

### Cedrol Arrested Cell Cycle at G1 Phase

**Fig. 3:**
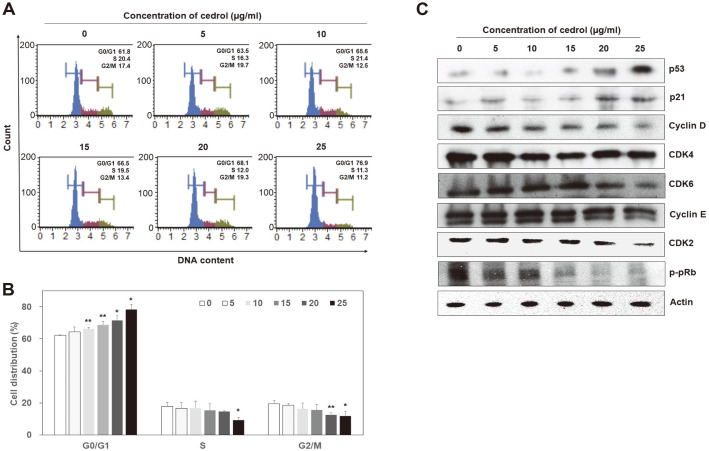
Cedrol induces G1 arrest of the cell cycle in A549 cells. (**A**) After treatment with indicated doses of cedrol for 48 h, cell cycle profiles were evaluated using a Muse Cell Cycle Kit with a Muse Cell Analyzer. (**B**) Percentages of cell populations in G0/G1, S, and G2/M phases are calculated for each dose of cedrol. Data are presented as mean ± SD of three independent experiments. *p < 0.05 and **p < 0.01 vs. each control. (**C**) Western blot analysis was performed after treatment with indicated doses of cedrol for 48 h to determine whether the expression of cell cycle-related proteins was altered by cedrol treatment. Actin was used as an internal control.

Cell cycle distribution was analyzed using a Muse Cell Cycle Kit to determine if cell cycle arrest was involved in the inhibition of proliferation of A549 cells by cedrol treatment (Figs. 3A and 3B). Cells were recovered after 48 h of treatment with cedrol (0, 5, 10, 15, 20, and 25 μg/ml). Quantitative analysis was performed for G0/G1, S, and G2/M phases using a Muse Cell Analyzer. Compared to control cells treated with 0.1% DMSO (62.10 ± 0.43), cedrol-treated A549 cells accumulated in the G0/G1 phase of the cell cycle significantly (*p* < 0.01) in a dose-dependent manner (5 μg/ml cedrol: 64.33 ± 3.04 ~ 25 μg/ml cedrol: 78.27 ± 3.27), resulting in a decrease in their S (5 μg/ml cedrol: 16.73 ± 3.70 ~ 25 μg/ml cedrol: 9.33 ± 1.70) and G2/M S (5 μg/ml cedrol: 18.47 ± 1.33 ~ 25 μg/ml cedrol: 11.73 ± 3.03) phases. These data suggest that inhibition of cell viability in A549 cells by cedrol is associated with the induction of G0/G1 arrest. Accordingly, to determine whether the expression of cell cycle regulatory proteins was altered, western blot analysis was performed after cedrol treatment for 48 h (Fig. 3C). During the G1/S transition period, CDK2 forms a complex with cyclin E and A and is activated, and the activated CDK2/cyclin E complex phosphorylates pRb to release E2F, a transcription factor necessary for S phase, to initiate the S phase. p21, a cyclin-dependent kinase inhibitor, is an important factor that regulates cell cycle progression by inhibiting the activity of the cyclin/CDK complex [27, 28]. As shown in Fig. 3C, the expression of p53 and p21 increased with the dose of cedrol, whereas the expression of the other cell cycle-related proteins tested, including CDK2, CDK4, CDK6, cyclin D, cyclin E, and phosphorylated retinoblastoma protein (p-pRb), decreased in a dose-dependent manner. These results validated the cedrol-induced cell cycle arrest at the G1 phase at the molecular level.

### Cedrol Induced Apoptosis

**Fig. 4:**
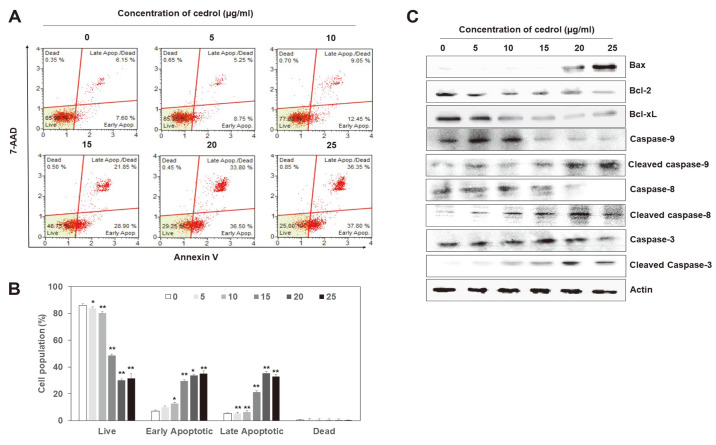
Cedrol induces apoptosis in A549 cells. (**A**) After treatment with indicated doses of cedrol for 48 h, cells were collected and stained with annexin V and 7-AAD. Induction of apoptosis by cedrol treatment was measured using a Muse Annexin V and Dead Cell Assay Kit with a Muse Cell Analyzer. (**B**) Percentages of early and late apoptotic cells were then calculated for each dose of cedrol. Data are presented as mean ± SD of three independent experiments. *p < 0.05 and **p < 0.01 vs. each control. (**C**) Effects of cedrol on the expression of apoptosis-related proteins were confirmed by western blot analysis after treatment with indicated doses of cedrol for 48 h. Actin was used as an internal control.

Phosphatidylserine (PS) is normally restricted to the inner membrane of the lipid bilayer. In apoptosis-induced cells, however, PS is exposed to the cell surface due to decreased viscosity, increased fluidity, and asymmetric loss of phospholipids in the plasma membrane [29, 30]. Annexin V is a cellular protein that binds to PS and is commonly used as an early marker for apoptotic cells [31]. The frequency of annexin V-positive cells was measured using a Muse Cell Analyzer to determine whether the effect of cedrol on A549 cells was associated with induction of apoptosis (Figs. 4A and 4B). The proportion of annexin V-positive cells in A549 cells increased significantly (*p* < 0.01) by cedrol treatment in a dose-dependent manner at the expense of live cells. In addition, early apoptotic cells (Annexin V+/7-AAD-) increased more than late apoptotic cells (Annexin V+/7-AAD+) upon treatment with cedrol. Overall, these results indicate that cedrol promotes apoptosis in A549 cells. We further examined the expression of apoptosis-related proteins in A549 cells treated with cedrol for 48 h by western blot analysis (Fig. 4C). Cedrol treatment increased the levels of the pro-apoptotic molecule Bax in a dose-dependent manner, whereas it decreased the expression of the anti-apoptotic molecules Bcl-2 and Bcl-xL. The levels of cleaved caspases-3, -8, and -9 increased in A549 cells treated with cedrol in a dose-dependent manner, whereas their inactive forms were decreased. Taken together, these results indicate that the cytotoxic effect of cedrol (Fig. 2B) is related to the induction of apoptosis in A549 cells.

## Discussion

Since DNA is replicated indefinitely in cancer cells that proliferate unlimitedly, proteins related to DNA replication have been proposed as cancer biomarkers. Among them, the MCM protein complex has been reported to be overexpressed in various cancer cells and has therefore drawn attention as both a biomarker for cancer diagnosis and a target for cancer treatment. Therefore, the molecules that inhibit the expression of MCM protein were screened in natural products, and *J. chinensis* was selected as a candidate. Bioassay-guided fractionation and isolation on *J. chinensis* was then performed to obtain an active single compound, which was identified as cedrol by mass and NMR spectroscopic analyses (Fig. 1). The w/w yield of cedrol in *J. chinensis* was about 1.24%.

Cedrol, a crystalline natural substance, is a bioactive sesquiterpene alcohol found in the essential oil of conifers, especially in the cypresses and *Juniperus* [32, 33]. Cedrol has been commonly used worldwide in decorative cosmetics, fine fragrances, shampoos, and soaps, as well as in non-cosmetic products such as detergents [34]. Several studies have demonstrated that inhalation of cedrol induces an increase in parasympathetic activity and a decrease in sympathetic activity, resulting in sedative and relaxant effects [35-38]. Furthermore, several studies related to the anticancer activity of cedrol have recently reported that cedrol inhibits proliferation of human amelanotic melanoma C32 and renal adenocarcinoma cells [39], induces autophagy and apoptosis in A549 non-small cell lung carcinoma cells [40], and suppresses glioblastoma progression by triggering DNA damage and blocking nuclear translocation of the androgen receptor [41]. In addition, cedrol was reported to chemosensitize cancer cells and suppress cell proliferation by destabilizing plasma membrane lipid rafts in human leukemia K562 and colon cancer HT-29 cells [42]. It has also been reported that *Pyrolae herba*, which contains cedrol, inhibits proliferation of human chondrosarcoma SW1353 cells [43]. However, there have been no reports that cedrol is involved in the expression of MCM proteins. Thus, this is the first study to report that cedrol inhibits the expression of MCM proteins and induces cell cycle arrest and apoptosis in A549 cells, and moreover, the first to provide information on cedrol-induced cell cycle arrest and apoptosis at the molecular level.

As is well known, specific cyclins are synthesized for each cell cycle to form a complex with CDK, and the cell cycle proceeds by the activated cyclin/CDK complex. Earlier in the G1 phase, CDK4 and CDK6 are activated by binding to cyclin D, respectively, to partially phosphorylate pRb. E2F, a transcription factor required for the S phase, is then partially activated by the p-pRb, leading to the progression of the G1 phase. Later in the G1 phase, cyclin E is synthesized and binds to CDK2 to form an active complex. During the G1/S transition period, the activated cyclin E/CDK2 complex phosphorylates pRb, releasing E2F to initiate the S phase. The CDK inhibitor p21, which is upregulated by p53, is an important factor in regulating cell cycle progression by inhibiting the activity of the cyclin/CDK complex [27, 28]. In the present study, cedrol strongly inhibited the expression of MCM2-7 and induced cell cycle arrest at the G1 phase and apoptosis in A549 cells (Figs. 2-4). It is believed that cedrol upregulates p53 to increase the expression of p21, which binds to the cyclin D/CDK4, cyclin D/CDK6, and cyclin E/CDK2 complexes and inhibits their activity, thereby inhibiting the phosphorylation of pRb. Consequently, downregulation of p-pRb reduces the release of E2F1, thereby inhibiting the progression of the G1 phase and entry into the S phase.

Several molecules that regulate MCM proteins and inhibit the proliferation of cancer cells, such as TSA and lovastatin (mentioned in Introduction), also induce cell cycle arrest and apoptosis in cancer cell lines [6, 8, 14]. The MCM proteins have been reported to be involved in the progression of cell cycle [6]. In MCM2 knockdown cells, the expression of pRb, cyclin D, cyclin A, and CDK4 decreased, p53 and p21 were upregulated, and cell cycle arrest at G1/S or G2/M phase was induced [7]. MCM3 knockdown resulted in G1 arrest by decreasing the expression of cyclin A [7]. MCM6 knockdown reduced the expression of CDK2, CDK4, cyclin A, cyclin B1, and cyclin E, and induced S/G2 arrest [9]. Taken together, it is suggested that cell cycle arrest by anticancer substances regulating MCM proteins is a result of the decrease in the expression of MCM proteins. Therefore, the results of this study indicate that the upregulation of p53 and p21 and downregulation of cyclin D, cyclin E, CDK2 and CDK4 by cedrol treatment is related to the downregulation of MCM proteins. The effect of cedrol is similar to that of widdrol extracted from *J. chinensis*, which was also previously reported by our team [24, 25]. Widdrol inhibited the expression of all MCM2~7, induced the expression of p53 and p21, inhibited the expression of CDK2, cyclin E, p-pRb, and PCNA, led to G1 phase arrest and apoptosis in A549 and HT29 cells. In addition, inhibition of MCM protein expression by widdrol in HT29 cells is associated with the activation of DNA damage checkpoint caused by DBS, leading to activation of ATM, p53, and p21 and reduction of cdc25a, CDK2, and cyclin E [26]. It should be noted, however, that unlike widdrol, cedrol did not induce DBS directly on DNA (data not shown). In addition, it is necessary to confirm whether the downregulation of MCM proteins by cedrol treatment is related to the activation of the JNK pathway.

Two different pathways inducing apoptosis are known, namely the intrinsic and extrinsic pathways that correlate with signaling types [44-46]. The intrinsic pathway is regulated by Bcl-2 family proteins which include pro-apoptotic and anti-apoptotic proteins. Cell survival and death are determined by the ratio between pro-apoptotic and anti-apoptotic molecules. When the intracellular balance is disrupted by a variety of cellular stresses, the expression of Bcl-2 family proteins is altered, mitochondrial membrane permeability and potential change, cytochrome c is released from the mitochondrial inner membrane, and the initiator caspase-9 is activated. In contrast, the extrinsic pathway is triggered by extracellular signals delivered in the form of death ligands binding to death receptors, which recruit adaptor proteins that bind to the initiator caspase-8 to form a death-inducing signaling complex. In each case of the intrinsic and extrinsic pathways, the executioner caspases-3, -6, and -7 are activated by the above-mentioned initiator caspases (*i.e.*, caspases-9 and -8 in the intrinsic and extrinsic pathways, respectively) to initiate cleavage of cellular proteins, leading to apoptotic cell death. In this study, the expression of the pro-apoptotic Bcl-2 family protein Bax increased in A549 cells treated with cedrol, whereas the expression of the anti-apoptotic proteins Bcl-2 and Bcl-xL decreased in the same cells. Furthermore, active forms of initiator caspases-9 and -8 and executioner caspase-3 were increased by cedrol treatment (Fig. 4C). These results suggest that cedrol activates both the intrinsic and extrinsic pathways to induce apoptosis in cells.

The MCM proteins have also been reported to be involved in apoptosis, and the expression of Bcl-2 has been reported to be related to the MCM expression [6]. These results indicate the alteration of Bcl-2 family expression by cedrol treatment in this study is related to the downregulation of MCM proteins.

**Fig. 5:**
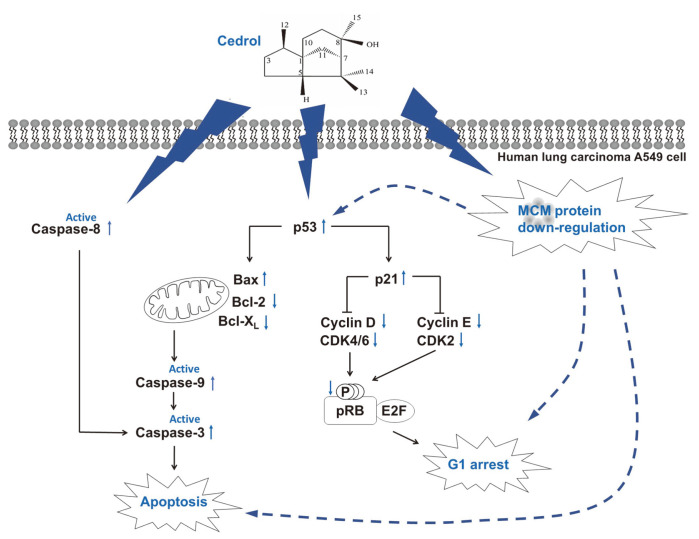
Proposed molecular mechanism of cedrol through G1 arrest and apoptosis association with MCM downregulation in A549 cells. Cedrol induces G1 arrest through the upregulation of p53 and p21, and downregulation of cyclin, CDK, and phosphorylated pRb. At the same time, cedrol induces apoptosis through the regulation of Bcl-2 family proteins and activation of caspase. Induction of G1 arrest and apoptosis by cedrol may be linked by its strong MCM inhibitory activity in A549 cells.

Taken together (Fig. 5), cedrol, a substance isolated from *J. chinensis*, strongly inhibited the expression of all MCM2~7 proteins in A549 cells. Like the results observed in several MCM knockdown cells [7, 9], cedrol induced the expression of p53 and p21, which inhibits the activity of G1 specific cyclin/CDK complexes, thereby inhibiting the phosphorylation of Rb and arresting the cell cycle at G1 phase in A549 cells. In addition, cedrol induced apoptosis through both intrinsic and extrinsic pathways, involving decrease of anti-apoptotic Bcl-2 and Bcl-xL protein expression, increase of pro-apoptotic Bax expression, and activation of caspase-8, -9, and -3. Induction of G1 cell cycle arrest and apoptosis by cedrol treatment in A549 cells is thought to be closely related to its MCM inhibitory activity.

The present study broadens our understanding of how cedrol executes its anticancer activity at the molecular level and demonstrates that cedrol is potentially applicable to the treatment of human lung cancer clinically as an inhibitor of MCM proteins. Further studies on cedrol will be needed in regard to elucidating the pharmacological mechanisms of its actions related to MCM proteins in detail and evaluating its anticancer activity in both various cancer cell lines in vitro and animal models in vivo.
